# Developing the Moti-4 intervention, assessing its feasibility and pilot testing its effectiveness

**DOI:** 10.1186/s12889-015-1826-y

**Published:** 2015-05-21

**Authors:** Hans B. Dupont, Paul Lemmens, Gerald Adriana, Dike van de Mheen, Nanne K. de Vries

**Affiliations:** Moti-4 Research Project Coordinator Maastricht University/VPN/Mondriaan, Waldeck Pyrmontsraat 53, 6224 LM Maastricht, The Netherlands; CAPHRI, School for Public Health and Primary Care, Maastricht University, Maastricht, The Netherlands; Addiction Prevention Department Mondriaan, Heerlen, The Netherlands; Addiction Research Institute, Rotterdam, The Netherlands; Erasmus MC, Rotterdam, The Netherlands; Health Promotion, CAPHRI, School for Public Health and Primary Care, Maastricht University, Maastricht, The Netherlands

**Keywords:** Intervention Mapping, Cannabis, Adolescents, Targeted prevention, Alcohol and drug prevention, Motivational Interviewing, Evidence-based interventions

## Abstract

**Background:**

The Moti-4 intervention was developed to prevent addiction and other health problems among vulnerable adolescent cannabis users. The aims of Moti-4 are to reduce the use of cannabis among adolescents and to encourage their motivation to change their behavior.

**Methods:**

Intervention Mapping, a systematic approach to developing theory- and evidence-based interventions, was used to develop a protocol for the intervention. The process of developing the intervention also used the method of responsive evaluation to explore the opinions of the immediate target group and intermediaries (N = 31). Feasibility was assessed in 9 interviews and analyzed in grids.

A quantitative pilot analysis involving a pre- and post-assessment in 31 subjects assessed whether the intervention was able to reduce drug use and would change intentions to change drug use behavior.

**Results:**

Using Intervention Mapping resulted in the development of a substantial four-session intervention with a clear manual and training for prevention workers. The choice of 12 consecutive steps was based on the Trans Theoretical Model of Behavior Change, Motivational Interviewing, Theory of Planned Behavior and the Self Determination Theory.

Positive aspects of working with Moti-4 were assessed in a feasibility study. Criticism by users has led to improvements to the manual.

In the pilot study, the average weekly amount spent on cannabis decreased significantly from an average €17.77 to €11,95 in the period after the intervention, with a medium effect size (*d* = 0.36). Likewise, a significant decrease was found in the frequency of use during the past week, from 4.3 to 2.4 (*d* = .52). As to motivation to change, a statistically significant increase was found in planning (*d* = .44) and a large increase in the desire to stop (*d* = .76). The change in the motivation to smoke less cannabis was small.

**Conclusion:**

Intervention Mapping proved to be a useful approach for the development of the intervention, using a productive combination of theory and community knowledge. The pre- and post-test pilot study showed that the intervention generally brought about a considerable positive change in the two principle targets, cannabis use and motivation. There is a need for further (controlled) research into its effectiveness and implementation as a standard method in addiction prevention services.

## Background

Cannabis is the most commonly used illicit drug worldwide [1]. Compared to other European countries, cannabis use in the Dutch general population holds an average position [2, 3]. However cannabis use among Dutch teenagers is substantially higher than in most other EU countries [4], with a prevalence among of use in the past month among 15 to 16 year olds of 14%, compared to 7% European wide [3, 4]. Since cannabis use is predominantly a problem of young people, public health concerns specifically regard the underage population. It is estimated that one in every ten users develops addiction problems [5]. An indicator of the severity of the problem is the trend in the number of registered patients in the Netherlands being treated for cannabis as a primary addiction problem, which has more than quintupled between 1994 and 2009 and doubled between 2006 and 2011 [3]. About 1700 (17%) of them were underage. The reasons for this increase are probably manifold [6]. Contributing factors that have been proposed include changes in use, a sharp rise in the THC concentration in cannabis between 2000 and 2006 (from 9% to 20%) [7], a change in the perception of cannabis use complications, and an increase in the number of treatment options.

Cannabis use is associated with a variety of psychosocial and health problems, including cognitive and respiratory impairment, psychotic episodes, and dependence [5, 8–11]. There is a clear link between early onset and subsequent low educational achievement [12]. A review of three studies concluded that early use of cannabis may contribute up to 17% to the rate of failure to achieve educational milestones such as high school completion, university enrolment, and degree attainment [10].

In the Netherlands, cannabis use is quite common in vulnerable groups. For example, the percentage that uses cannabis in the past month among school drop-outs was found to be 55%), up to 87% in marginalized and homeless youths, 59% in adolescents in custody, 45% in youths in truancy projects, and 45% in children of mentally ill or addicted parents, and youths attending special education [3, 13]. Henry [14] found full-day truants to have a three times higher prevalence of past-month use of cannabis than non-truants (0.49 vs. 0.15). In the Dutch province of Limburg, several ‘rapid assessment response’ (RAR) studies have been conducted, focusing on young loiterers in lower-income neighborhoods [15]. In ten neighborhoods, almost half of the target group of loiterers aged 13 to 23 years (N = 391) were actual cannabis users.

Prevention efforts have grown along with the growth of formal treatment options. A recent Dutch survey found no less than 243 alcohol and drug prevention interventions available for adolescents aged up to 23 years [16], some active, though most inactive, and of varying quality. In recent years, prevention campaigns have increasingly shifted from universal to more specific selective and indicated prevention efforts (such as brief interventions), with approaches targeting high-risk groups. There is a general consensus that treatment is distinguished from brief preventive interventions by the length of sessions: more than five 1-h sessions are regarded as treatment [17]. Brief interventions focus on increasing insight and awareness regarding substance use and on motivation toward behavioural change [17].

Interventions targeting vulnerable groups have become increasingly important in addiction prevention [15, 18, 19]. A general conclusion of the survey referred to above [16] was the need to fill the gap between more formal, high threshold treatment approaches and general prevention. It was concluded that no evidence-based intervention for specific risk groups was available in the Netherlands.

In an attempt to fill this gap, a randomized controlled trial of an Australian two-session intervention called the Adolescent Cannabis Check-Up (ACCU) [20] was recently tested in a Dutch sample [21]. The ACCU was translated into Dutch as “Weed-check”. The positive Australian results [20] were not replicated in the Netherlands [21]. It was conjectured that the “strait-jacket” character of the ACCU partly explains this discrepancy. The strict requirements in ACCU regarding fidelity to the protocol do not allow the interventionist to adapt and modify the intervention according to the client’s individual needs, and would thus limit its feasibility in a Dutch context Moti-4 is an attempt to construct an alternative approach which is more applicable to the Dutch situation. This article describes the development of this brief intervention for adolescent cannabis users, as well as an assessment of its feasibility, and a pilot study of its effectiveness.

## Methods

### Intervention mapping

As noted above, a major gap in the arsenal of existing interventions was that of interventions reaching out to non-treatment seeking adolescent cannabis users. This need led to the development of Moti-4, an intervention primary aimed at reducing cannabis use among adolescents in order to prevent drug dependency and related mental and physical problems. Intervention Mapping was used to guide this process.

Intervention Mapping [22] is a protocol designed to guide the creation of health promotion programs. The Moti-4 program was developed following the six consecutive steps of Intervention Mapping: needs assessment; behavioral and learning outcomes; selecting methods of behavior change and translating methods into intervention strategies and materials; producing the program components; program adoption and implementation; and planning of program evaluation.

Moti-4 is intended for cannabis users in adolescence (ages 14–24 years). Adolescents are referred to the prevention service by their parents, by agencies for youth care and drop-outs, and by student counselors in the school system.

The target group includes only actual users (i.e. past-month users), who additionally show one or more signs of problematic use.

Signs of emerging problematic use include:

- A clear causal link between cannabis intake and problems at school, work or in relationships [12, 14].
- Physical or mental health problems [11, 23].
- Vulnerabity for developing problematic use because of some predisposition, such as marginalized youths, truants, children of addicted parents, and young people attending special education [3, 13].
- Experimenting in a way that does not befit the adolescent’s age. This is the reason why the inclusion criterion for young adolescents (<18) is any cannabis use, while the criterion for the older adolescents (>18) is high-dose cannabis use [12].

The intervention is appropriate for youngsters with different cognitive capacities/IQs, ethnic backgrounds and/or family circumstances. Next to outcome parameters pertaining to cannabis use, specific outcomes also include intermediating factors. These are in part determined by theories of behaviour change.

Moti-4 is based on the Theory of Planned Behavior [24], and its later adaptations [25]. This theory postulates that intention, the most important determinant of behavior, is in itself determined by three independent constructs: attitude, subjective norms, and perceived behavioral control. Motivation is of major concern in this intervention. Knowledge of the effects of cannabis and dependence form the basis for change [24]. Problem awareness is raised and behavior resilience and engagement in behavioral alternatives are encouraged. Influencing the social norms of the adolescents’ environment is expected to reinforce the desired behavior [22, 24]. The theoretical basis of Moti-4 also includes Self-Determination Theory (SDT), in which conditions supporting the individual experiences of autonomy, competence, and relatedness are argued to foster the most volitional and most effective forms of motivation [26].

A second objective of the Moti-4 intervention aims to encourage and motivate the adolescents to change their intentions toward use [20, 21, 27–29]. Guiding principle here was Motivational Interviewing (MI), which is a client-centered counseling style that aims to explore and resolve ambivalence about changing personal behaviors [27], based on the Trans Theoretical Model of Behavior Change [28].

The intended engagement is a preventive intervention. Further treatment and care are normally beyond the scope of Moti-4, but by means of a careful screening, adolescents with severe drug-related problems have to be stimulated to look for professional care and treatment [30].

In four sessions, Moti-4 aims to stimulate youngsters to critically examine and adjust their own drug use. This is achieved by screening for substance use and problems in related areas such as problems at school or in the family, or psychiatric or physical problems [30, 31], followed by knowledge transfer, creating awareness, motivational interviewing, and strengthening the youngster’s resilience. Should the prevention worker experience a sense of alarm during the screening, and he ascertains that the adolescent has a substance abuse disorder, he or she will be referred to treatment as soon as possible [30].

### Responsive evaluation

After the four sessions had been clearly described in a manual, the opinions of the target group and intermediaries using the responsive evaluation method were explored [32]. Information sessions were organized, involving stakeholders, among whom addiction prevention workers, health care workers, social workers, community workers and members of the immediate target group (N = 31). In the sessions a social climate was created in which the stakeholders would feel free to express their commitment to, but also concerns about the proposed intervention. An outcome of this was that, contrary to results of a literature review that had emphasized motivational interviewing, feedback, advice, and strategies to reduce consumption [20, 29, 33–48], the health care workers who were interviewed rather focused on screening, knowledge, risks and alternatives, gaining trust, and motivating. A further suggestion from the responsive evaluation was to prevent no-shows by visiting the youngsters on site. The adolescents interviewed considered the Moti-4 meetings to be useful. They were able to remember essential parts of the program, but found it difficult to change their behavior. Prevention workers and social workers indicated that Moti-4 would have to be implemented by means of a protocol (which can be used as an instruction manual), peer review meetings, and publicity, in order to enhance awareness of the program. The Moti-4 manual was then adapted, based on the results of this responsive evaluation.

A crucial element in the use of Moti-4 is a 14-item checklist (Table 1). Each item must be dealt with in a consecutive order during the four meetings. Several tools for all 14 items are available in the Moti-4 manual. For instance, the item of *knowledge transfer* can be addressed using various quizzes and/or videos, depending on the level of the adolescent and the personal preference of the prevention worker. Adherence ot the protocol of the 14 items is considered essential.

**Table 1:** The 14 items discussed during each of the 4 sessions in the Moti-4 intervention, their purpose and the number of the session in which the item is most important

Obligatory items	Purpose	session #
Assessment of use and life areas (e.g. euroADAD, MATE-Y)	Triage, screening [30, 31]	1
Stage of use	Triage, indication [28]	1
Recording use/diary	Self-monitoring [47]	1/2
Users chart	Monitoring, social norm [47, 48]	1/2
Knowledge transfer	Increasing knowledge [24]	2
Reasons for use	Motivational interviewing [27]	2/3
Pros and cons balance	Motivational interviewing [27]	2/3
Confidence measuring rod	Readiness for change [27]	3
Social network	Social norm; relatedness can foster effective motivation [49]	3
Peer pressure and craving	increasing relatedness and resilience [49]	3
Plan for change	Action planning, coping planning, self-monitoring [47]	2/3
Feedback given to referring person	Relatedness, support [49]	4
Meeting with parents or educators (optional)	Relatedness, support [49]	4
Planning follow-up	Sustainability	4

Recruitment for the intervention focuses on finding vulnerable adolescents such as marginalized youths, truants, children of addicted parents, and youths attending special education. Adolescents are referred by their parents, by agencies for youth care and drop-outs, and by student counselors in the school system.

### Feasibility study

In 2012, the intervention was adopted by four of the twelve large regional addiction treatment institutes that almost monopolistically provide all substance abuse treatment and (secondary) prevention activities in the country. Thirty experienced prevention workers (with a degree form a university of applied sciences) had been trained by the first author and an assistant to carry out Moti-4 for a pilot study. Previous training in motivational interviewing was a precondition for taking part. The training course involved ‘going through the motions’ for the four sessions, the use of the tools, recruitment of the target group members, the theoretical background of Moti-4, and an explanation of the need for evaluative research.

So far, 102 prevention workers in 7 provinces in the Netherlands have been trained in the use of this program. Trained prevention workers carrying out Moti-4 have to put great effort into actively finding these vulnerable participants, and in teaching intermediaries how to recognize and refer them. The feedback and the experience of these colleagues will be used to further update the Moti-4 manual.

After the training course, a feasibility check was performed among a convenience sample of nine prevention workers. At that time, each worker had recruited at least two adolescents for the Moti-4 intervention. Five trainees were male, four were female, and their age ranged from 25 to 46 years. They were interviewed about their experiences with the program items and with recruiting adolescents from the target groups, and about the need for further assistance. The interviews took 30–60 min and were analyzed in grids, using a protocol based on the RAR methodology [49].

A major topic that emerged in the interviews was that of adolescents dropping out of the program. The attrition rate during the pilot study was estimated to be 30 %; adolescents dropping out after the second, third or even fourth session. According to respondents, reasons for dropping out were the severity and nature of the problems these youngsters were dealing with, and the fact that attending four sessions was too much of an effort for them. Several youngsters who were initially thought to be eligible were found not to have cannabis as their primary issue, but gaming, alcohol or the use of other substances. For this reason, eligibility criteria were later tightened, after no more dropout were recorded in the pilot study.

The interviewed staff rated working with Moti-4 as satisfactory. Favorable aspects of Moti-4 that were mentioned included the logical composition and the clear structure, the large choice of easy-to-use tools, the broad applicability (also valuable for other issues and different problem levels), the involvement of parents and other educators, and the fact that the intervention concludes with a concrete plan. Furthermore, appreciation was expressed for the relative freedom in the protocol to choose a tool that is suitable to the particular adolescent, the personal preference of the prevention worker, and the local organizational framework, particularly in comparison to other interventions such as ACCU [20]. They confirmed that experience with motivational interviewing was indeed necessary.

The staff were less enthusiastic about the methods used for the primary screening, i.e. euroADAD and the Dutch MATE-Y Questionnaire, which they regarded as too elaborate. They questioned the added value for clinical practice. They also expressed concerns about Moti-4 being less suitable for low-skilled adolescents. Constructive feedback was also given on the tools to promote readiness for change (confidence measuring rod and plan for change) used in the third session.

### Pilot study

The design was a pre- and post-assessment among the 31 non-treatment seeking adolescent cannabis users enrolled in the intervention. The aims of Moti-4 are to reduce the use of cannabis among adolescents and to increase their motivation to change their behavior. The primary outcome measure in the pilot was the quantity of cannabis use in the week, covered by the question to estimate the amount of euros per week spent. If the youngster is growing cannabis himself or gets it for free he/she is asked to give a reliable estimation. We consider the amount of money spent the most reliable estimation in a self-report. Yet, as a reliability check, the questionnaire also asked the respondent to estimate the number of ‘joints’ they had smoked.

The second set of parameters concerned behaviorial determinants, covered by 24 questions on perceived behavioral control, social norm, attitude, and intention to change.

The psychosocial determinants (i.e. attitude, perceived behavioral control, social influence, intention and action plans) of cannabis use were based on the I-Change model [25]. All determinants were assessed with items using five-point Likert answering scales, which were later combined into one variable for each determinant. Attitude was measured with eight items, four of which regarded the pros and four the cons of cannabis use. Perceived behavioral control was assessed by two questions (*How difficult is it for you not to smoke cannabis?; How difficult is it for you to refuse a joint when a friend offers you one?*). Social influence was assessed by one social modeling question, four questions on social norm and one question on perceived peer pressure. Three kinds of intentions were measured: the intention to use cannabis, the intention to quit and the intention to reduce cannabis consumption. Action plans were measured by three questions.

The reliability of the resulting attitude scale was modest at entry (α = .60), but had increased slightly at the second measurement (α = .69). The associations between the three items covering positive expectations did not warrant scaling, with low α-values at both instances. Two self-mastery items were combined (α = .75), to indicate how well the respondent was able to withstand internal and external urges to smoke cannabis. Four items on positive social norms were combined, as were four items on negative social norms. Both scales had low internal consistency (average at two time points .35 and .53). Remarkably, an increase in the internal consistency of the instruments was observed in assessments after the intervention. This could point to either a decrease in the homogeneity of the sample or to real change as a result of the intervention.

Additional data were collected using a self-report questionnaire consisting of seven items on socio-demographic data, including gender, living situation, level of education, nationality, and country of birth of mother and father.

The sample included 31 non-treatment seeking adolescents aged 14 to 24 years (M = 17.9) with recent cannabis use (at least once last month). They were the first persons (convenience sample) to be recruited by twelve prevention workers, from four prevention agencies. These prevention workers assessed their eligibility (recent and potential problematic use, vulnerable background). Twenty-two of the participants were male (71%). Their education level was low (77.4% lower vocational education). Almost half of the participants had at least one parent not born in the Netherlands (n = 14).

Moti-4 was administered at the offices of the prevention agencies. Participants completed the four sessions within a month, with at least a one-week interval between the sessions. Participants filled in the questionnaire just before and a week after the intervention. All participants signed an informed consent form before participating. They received vouchers as compensation for their time.

## Results

After the intervention, the average weekly amount spent on cannabis had decreased from €17,77 to €11,95 (*p* < .05; see Table 2), with a medium effect size (*d* = 0.36). Likewise, a significant decrease was found for the past week’s frequency of use, from 4.3 to 2.4 (*d* = .52). As regards the motivation to change, a statistically significant increase was found for planning (*d* = .44) and a large increase in the desire to stop (*d* = .76). The change in the motivation to smoke less cannabis was small. Negative effect expectancies had increased significantly after the intervention (*d* = .48). Positive expectations were generally neutral and did not change during the intervention. Some lasting changes were observed. Initially (at pre-test), the relaxing effect of cannabis was positively related to negative expectations (*r* = .42). At the post-test, a negative attitude was positively associated with low social benefits of smoking (*r* = .40).

**Table 2:** Results pre- and post-test pilot study Moti-4

N = 31		Pre-test Mean (SD)	Post-test Mean (SD)	Dependant *t*-test value (df)
	€’s cannabis per week	€17,77 (17)	€11,95 (15.5)	t(30) = 2.15^a^
	Past week’s frequency of use	4.3 (4.2)	2.4 (3.0)	t(30) = 2.69^a^
	Daily (tobacco) cigarettes	13.9 (17.6)	15.3 (17.4)	t(29) = −1.20
	Negative attitude (school/work ; lungs ; psych.; money).	14.5 (2.9)	15.9 (2.5)	t(30) = −3.38^a^
	Positive attitude (social; happy; relaxed)	10 .8 (1.7)	10.5 (1.8)	t(30) = .95
	Self efficacy	5.2 (2.3)	6.7 (2.1)	t(30) = −4.48^b^
	Social norm (negative)	11.9 (2.8)	12.1 (2.5)	t(30) = −.486
	Social norm (positive)	10.6 (2.3)	10.7 (2.9)	t(30) = .29

^a^P < 0.05; ^b^P < 0.001

Self-mastery increased significantly during the intervention (p = .000; *d* = .51). Positive social norms conducive to smoking did not change (*r* = .57), nor did the negative social norm scores (indicative of a negative norm toward cannabis use). The correlation between positive and negative social norms did, however, increase over time in terms of absolute magnitude, from *r* = −.53 to *r* = −.69.

## Discussion

The results show that the intervention generally brought about a considerable positive change in the two principal targets, viz. cannabis use and motivation. These changes were accompanied by similar changes in some determinants of behavior, viz. attitude and self-mastery. No clear changes were found in a third factor, that of social norm. This may be due to the short time frame in which both the intervention and its evaluation took place. This might also explain the absence of effects in terms of perceived social norms. Perceived use and the influence of friends make up a large part of what is regarded as (descriptive) norm or (prescriptive) normative rules. These norms and normative perceptions change only gradually and, may depend on the choice of an adolescent’s social network.

The evaluation of the results of the pilot study using a trial version of the Moti-4 has led to the implementation of the Moti-4 intervention on a larger scale. Meanwhile, a study is being conducted including 27 prevention workers and 124 adolescents, to make the findings more robust (Trial registration number RCT; METC Atrium-Orbis-Zuyd: 12-N-110). In both this RCT and beyond, attention is being paid to a more effective recruitment protocol. Although it is important to maintain the necessary manoeuvring space for intervention workers with regard to recruitment (which was regarded as a positive aspect of Moti-4 in the responsive evaluation by users), inclusion of severe cases or persons with multiple problems or psychiatric disorders should be avoided. The RCT involves an assessment of the determinants of dropout.

The experiences from the feasibility check and the pilot study have led to adjustments to the protocol eventually used for the RCT. Alternatives to the screening instruments (such as euro-ADAD and MATE-Y) have been added to the manual (a semi-structured questionnaire). Eligibility criteria have been tightened, and also tools have been added that can be used for very low-skilled adolescents. In their recent edition of MI [27], Miller & Rollnick discouraged the use of the decisional balance. Nevertheless, it is regarded [27] as a potentially useful instrument when a client is in an early stage. Future versions of the Moti-4 handbook will take this into account.

An unexpected finding in our pilot study was that the motivation to smoke less cannabis did not seem to change, while the actual use did. The Transtheoretical Model of behavioural change, also known as the Stages of Change (SOC) model [28] has been widely criticized in recent years [50]. An interesting future research question would be if the process of behaviour change follows the SOC model in Moti-4 participants. Based on our results we hypothesize that self-mastery plays an important role.

Since one of the goals of effective public health research is “filling the toolbox”, it would be interesting to assess the suitability of Moti-4 in comparison with similar interventions [29]. Addiction Prevention Netherlands (VPN), an alliance of the official professional alcohol and drug prevention agencies in the Netherlands, is trying to establish a national minimum set of evidence-based interventions [16]. Moti-4 has been developed with the aim of becoming one of these nationally available VPN interventions, once its effectiveness has been established.

Finally some limitations must be taken into account. This is a pilot study. To make these findings more robust, a larger N is needed. This research totally relied on self-reports. Having four sessions, Moti-4 respondents might have developed an intensive contact with the prevention worker, resulting in more social desirability in the answers of our Moti-4 respondents. Social desirability might also be aroused by the fact that participants were referred by teachers, youth-workers and parents. Cross checking with blood test might solve this problem.

## Conclusion

The above evaluations indicate that within a coherent public health policy, an intervention like the Moti-4, aimed at a reduction of cannabis consumption in adolescents, could be a useful addition to the existing arsenal of interventions, though further research is needed to make the findings of this pilot more robust.

The Intervention Mapping method proved satisfactory in streamlining the process of developing the Moti-4 intervention. Well-designed and effective interventions should be guided by theory and informed by empirical evidence regarding change targets. Intervention Mapping has helped us to develop a well-founded program using an iterative process. The systematic approach adopted was a productive combination of theory and community knowledge, which finally resulted in the Moti-4 program.

In the process evaluation, the prevention workers interviewed reported positive aspects of working with Moti-4. They appreciated the logical composition and the clear structure, the wide choice of easy-to-use tools, the broad applicability, the choice to involve parents and other educators, the fact that the program concludes with a concrete plan, and the freedom afforded by the protocol to choose tools adapted to the level of the adolescent and to the personal preference of the prevention worker. Concerns were expressed by the professionals about the complexity of the euroADAD, and MATE-Y screening instruments, and the applicability of Moti-4 for very low-skilled adolescents.

Quantitative analyses show that the intervention results in less use of cannabis and an increased motivation to change. Self mastery changes significantly as a result of our intervention.

Successful Implementation of the program is demonstrated by the large number of prevention workers who have now been trained in performing Moti-4. Although Moti-4 has already become well-known nationwide, further research into its effectiveness and the feasibility of wider implementation to problematic use of other substances remains necessary.

### Ethical approval

Ethical approval for this study was granted by METC: 12-N-110 (Medisch Ethische Toetsings Commissie Atrium Heerlen).

## Abbreviations

IM: Intervention Mapping
MAP: Mondriaan Addiction Prevention
MI: Motivational Interviewing
RCT: Randomized Clinical Trial
SDT: Self Determination Theory
TPB: Theory of Planned Behavior
VPN: Addiction Prevention Netherlands
